# Progress in Surgery

**Published:** 1900-07-21

**Authors:** 


### PROGRESS IN SURGERY.

### OBSTETRICS.

(*Continued from page* 241.)

**The Vomiting of Pregnancy.** — Evans,[21] reasoning from two cases of vomiting during pregnancy in which he noticed that attacks of vomiting were induced during examination of the breasts and the vagina, as well as a distinct rhythmical tendency in their attacks and the greater severity during the menstrual epoch, holds that there must be a rhythmical cause for the condition. This he finds in the rhythmical contractions of the uterus during gestation, which perhaps are due to effete matters in the sinuses of the uterus urging a reflex contraction. Thus, in the morning on rising determination of blood to the uterus leads to energetic contractions, and so to vomiting. Give the stomach active work previously and the contractions are lessened, because less blood reaches the pelvic organs. Nerve sedatives act by controlling reflex irritation.

**Puerperal Infection.**—Macharg[22] has published an analytical account of 57 cases, of which 31 ended fatally, while 26 recovered. Primiparæ were often affected, while it occurred not rarely after abortion or premature labour. The injuries due to the labour were anything from a slight wound to an extensive laceration. The onset was the commonest from first to fourth day, generally second. Some were delayed to seventh day or longer. A fortnight was the usual duration, the sixth day being the earliest at which death occurred. There was no regularity about the spread of the inflammatory infection—there did not seem to be more inflammatory reaction round an injury. Enteritis was a common complication, sometimes with ulceration, one case markedly so. The bacteriological investigation was incomplete; in nine cases where streptococci were found the antitoxic serum was tried, but only in one case did improvement seem to follow. The routine treatment adopted was the packing of the uterine cavity with antiseptic gauze, with the antiseptic douch twice daily. The use of quinine was not encouraging. Complications, such as suppurating joints, were met as they arose.

Webber[23] communicates a case of well-marked puerperal septicæmia where one injection of 10 cc. of anti-streptococcic serum was followed by a marked improvement. The temperature fell two deg., the vaginal discharge, which had been foul, rusty-brown, and pungent, became cleaner and purulent. The patient made good her recovery, but for some time had a weakened mental condition.

Anderson[24] also quotes a marked improvement with the use of the antitoxic serum in a pronounced case of puerperal septicæmia. He injected 10 cc. five times at 24 hours interval, stating that the depressing effect was so marked that he dare not give it more often as advised.

Lyle,[25] speaking of the treatment of puerperal sepsis by means of the antitoxic serum, expresses his firm belief that no good had ever been obtained from it, but that it rather did harm. He urges most strongly the utmost surgical cleanliness in all labours: The parts to be cleaned; the routine douche avoided, especially with perchloride of mercury; aseptic instruments; the disuse of lubricants, except as a protection to the finger from gonorrhœa, &c.; the limitation of vaginal examinations; the immediate suturing of all perineal lacerations.

Tuffier and Bonamy[26] give indications for hysterectomy in puerperal infection: (1) Total retention of placenta when neither curette nor finger can bring it away. (2) When indications for Cæsarian section and sepsis coexist. (3) When curettage produces no improvement in cases of sepsis, such as when the temperature still rises. But he urges that the sepsis must be known to come from the uterus, or there may be acute septicæmia or pyæmia, general peritonitis, or the patient's condition may be too extreme.

21 Brit. Med. Jour., Apr. 28. 22 Brit. Med. Jour., Feb. 17. 23 Brit. Med. Jour., Feb. 17. 24 Brit. Med. Jour., Feb. 17. 25 Brit. Med. Jour., Mar. 24. 26 Internat. Med. Jour., Dec., 1899.

